# Changing environmental conditions impact the phenotypic plasticity of *Carex acuta* and *Glyceria maxima*, two common wet grassland species

**DOI:** 10.3389/fpls.2025.1542907

**Published:** 2025-04-28

**Authors:** Keith R. Edwards, Bernhard Glocker, Jiří Mastný, Tomáš Picek

**Affiliations:** Department of Ecosystem Biology, Faculty of Science, University of South Bohemia, České Budějovice, Czechia

**Keywords:** allometry, biomass allocation, coexistence, niche differences, phenotypic plasticity, wet grasslands

## Abstract

**Introduction:**

Maintenance of species coexistence is an important and on-going subject of plant ecology. Here, we aimed to determine how *Carex acuta* and *Glyceria maxima*, two common, co-occurring plant species in European wet grasslands, respond to changing environmental conditions and what these changes portend for coexistence of these two species. Such studies are important for predicting and modelling the effects of management and climate change on wet grassland plant species composition and for maintaining the ability of wet grasslands to provide their important ecosystem services including carbon sequestration and water purification. Based on past studies, we hypothesized that both species would be affected by hydrologic changes but that these effects would be modified by nutrient conditions with fertilization having a more positive impact on *G. maxima*.

**Methods:**

We established a mesocosm to distinguish the effect of hydrology and nutrients on the biomass allocation patterns of these two species to determine how environmental conditions may impact the life history traits of these two species, which would influence their ability to co-exist. Plants were grown in pots from late May to early September 2019 and subjected to two nutrient and three water level treatments. Half of the plants were harvested in July while the other half were harvested in early September and their biomass allocation patterns calculated. Univariable and multivariable analyses were conducted to determine the effects of the environmental treatments on the measured parameters. In addition, we determined the phenotypic plasticity of the two species and whether these showed allometric relationships to plant size.

**Results and discussion:**

*C. acuta* was affected more by hydrologic changes, growing better in dry and saturated conditions, while fertilization had a more positive effect on *G. maxima*. Both species were stressed when flooded, but *C. acuta* more so than *G. maxima*. Contrary to our predictions, *C. acuta* produced more ramets and was taller than *G. maxima*. Both species showed plastic responses to changing nutrient and water conditions, but only some were related to plant size. Our results indicate that *C. acuta* and *G. maxima* are more likely to co-exist in oligo- to mesotrophic wet grasslands with fluctuating water levels.

## 1 Introduction

Wet grasslands are semi-natural, graminoid-dominated habitats that are maintained through some sort of disturbance, usually grazing or mowing (Tallowin and Jefferson, 1999; Joyce, 2014). These grasslands can be highly diverse ecological systems that usually are found in agricultural landscapes, especially in Europe (Garcia, 1992; Klimkowska et al., 2007; Zelnik and Čarni, 2013). Both natural wet grasslands, such as those associated with prairie potholes, or those created and maintained by human activities, have similar hydrologic characteristics, being flooded periodically or having a high-water level, which influences their plant species composition (Joyce et al., 2016). Mowing provides not only fodder or bedding for livestock (Tasset et al., 2019), but, with the removal of plant biomass, allows for less-competitive plant species to survive and co-exist with more competitive species (Berg et al., 2012; Tardella et al., 2020).

Wet grasslands provide many important ecosystem services including nutrient removal, carbon sequestration, various hydrologic services such as flood attenuation and groundwater recharge, being important bird habitats (Joyce, 2014; Manton and Angelstam, 2021), as well as having a special microclimate that, through the cooling effect of evapotranspiration, can impact on the local and regional climate (Harding and Lloyd, 2008; Dietrich and Behrendt, 2022).

Native to Europe and Asia, *Carex acuta* and *Glyceria maxima* are two common plant species in Central European wet grasslands (Grootjans and ten Klooster, 1980; Hroudová et al., 1988; Nurminen, 2003; Tylová-Munzarová et al., 2005). *G. maxima* is considered to be an invasive species in North America, Australia and New Zealand, and South Africa (Mugwedi et al., 2014) where it may suppress native wetland plant species and affect the hydrology of whole wetland ecosystems (Sorrell et al., 2012). Therefore, it is important to study changes to the life history characteristics of these two plant species in more detail to understand how they spread, co-exist or outcompete other plant species.

Both species are emergent macrophytes which can form extensive, monospecific stands on gleyed clays or waterlogged organogenic soils (Hroudová and Zákravský, 2002). *C. acuta* is considered to be a slower growing, more conservative species which allocates more biomass to belowground structures, while growth of *G. maxima* is more in keeping with a competitive strategy, seemingly doing better in nutrient-richer conditions and allocating more biomass to aboveground structures. Both species are tolerant of moist conditions, as indicated by them having mean Ellenberg indicator values for moisture of 8 (Chytrý et al., 2018), but the species differ in their response to nutrient conditions, with *G. maxima* being associated with nutrient-rich wetland habitats while *C. acuta* is restricted to nutrient-poorer areas (mean Ellenberg nutrient values of 9 and 4, respectively). Prach (1993, 2008) found that *C. acuta* will replace *C. nigra* in formerly oligotrophic wet grasslands subject to fertilization, but it is outcompeted by reeds, including *G. maxima*, with increasing eutrophication, in agreement with the Ellenberg values.

However, there are situations when these two species can co-exist in the field (De Deyn, 2017; Edwards and Čížková, 2020; Glocker et al., 2024). Under what environmental conditions such long-term co-existence can arise and be maintained, and how changes in life history characteristics underlie this co-existence, is an important and on-going topic of plant ecology (Chesson, 2018; Bowler et al., 2022). Co-existence is thought to occur when two or more species occupy different niches, which may arise due to the partitioning of resources or by spatial and/or temporal heterogeneity (Chesson, 2000; Silvertown, 2004; Adler et al., 2010).

Edwards and Čížková (2020) proposed that spatial and/or temporal differences in site hydrology and nutrient availability would likely lead to *C. acuta* and *G. maxima* co-existing in wet grasslands. These authors predicted that co-existence of *C. acuta* and *G. maxima* would occur under moist, but not long-term flooded, conditions or in nutrient-rich but dry (un-flooded) habitats (Edwards and Čížková, 2020). However, it is difficult to separate the effect of these environmental factors in the field. In addition, their study only considered aboveground production and nutrient contents, but did not include belowground structures, nor did they take into account biomass allocation patterns or phenotypic plasticity, two parameters that are important in determining a species niche (Bowler et al., 2022).

Here, we established a mesocosm experiment to determine the effects of different nutrient and water level conditions on the growth of *C. acuta* and *G. maxima*, in particular the biomass allocation patterns of the two species. Our study thus expands on the study by Edwards and Čížková (2020) in order to answer some of the open questions left by their earlier, more limited study. In addition, we wanted to determine whether this plasticity is related to plant size, in other words whether there was an allometric relationship inherent in the observed biomass allocation patterns and vegetative spread of these clonal species (Jameson et al., 2022). Based on the literature and our past results (Edwards and Čížková, 2020), we predicted that 1) *G. maxima* plants would have greater biomass in nutrient-richer conditions, with greater allocation to aboveground structures, compared to *C. acuta* but 2) that this nutrient effect would be altered by hydrology thus indicating important interactive relationships between these two environmental factors. Additionally, 3) allocation patterns and vegetative reproduction would be dependent on plant size with larger plants having a greater number of new daughter shoots, being most notable in *G. maxima*. Therefore, based on these predictions, 4) *G. maxima* should be the more plastic species compared to *C. acuta* and should be favored over a wider range of environmental conditions.

## 2 Methods

### 2.1 Mesocosm set-up

Details of the mesocosm set-up are given in Glocker et al. (2024). Briefly, plants of *C. acuta* and *G. maxima* were collected from monospecific patches in the same wet grassland that was the focus of the study by Edwards and Čížková (2020). Plants, consisting of 2-3 shoots with attached roots, were transplanted into pots (9×9×14 cm; L×W×D) which were filled with a 2:1 sand/peat (by volume) mixture. Soil inocula were added to each specific pot using soil collected from patches of the two species which were assumed to contain the soil microbiome for each species. Pots with plants were then randomly distributed to different basins (187×120×45 cm; L×W×D) in order to better control water and nutrient levels. Thus, a basin would contain either *C. acuta* or *G. maxima* but never both.

The experiment consisted of a split-plot design with water levels nested within the nutrient treatment. Nutrient treatments were assigned randomly to each basin. Pots within a basin either received no added nutrients or were fertilized with 350 kg NPK fertilizer ha^-1^ yr^-1^ of an inorganic solution (Lovofert 15:15:15 NPK, Lovochemie, a.s.), added in two half-doses (mid-May and mid-July). The amount of added nutrients is the mid-point of the fertilizer application range recommended by the agri-chemical company. A micronutrient solution (“BioNova MicroMix”, BIONOVA, CR) was applied to the leaves of all plants at two-week intervals to insure that there was no micronutrient limitation. To minimize the chance of nutrient leakage from the pots, all pots were maintained at the low water level for three days to allow for nutrient mineralization and plant uptake. Also, past studies with *C. acuta* (Edwards et al., 2023, 2025) found rapid nutrient uptake by the plants. Therefore, any nutrient leaching from the pots to the water was assumed to be minimal. The pots within each basin were then subjected to three different water levels (dry = -15 cm below the soil surface; saturated = water level maintained at the soil surface; flooded = 15 cm above the soil surface) using wooden constructions. Overall, there were eight pots with plants per basin for each water level treatment for a total of 24 pots per basin. There were three replicate basins for each treatment combination (species × nutrient with water level nested within each basin).

### 2.2 Plant measurements

The plants grew from late May to early September 2019. During this time, plant height (height of longest leaf) and shoot number were measured at two-week intervals. Half of the plants were harvested in mid-July while the remaining pots were harvested in early September. Aboveground structures of the harvested plants were divided into leaves and stems, while belowground structures were carefully cleaned of soil and then separated into roots, rhizomes and rootstocks. In this case, rhizomes represent underground stems which lead to the production of new aboveground shoots, while the rootstocks are the belowground portions from which aboveground shoots arise, but not including the underground stems. Rhizomes were separated from the rootstock since rhizomes may be important storage structures which may have different nutrient composition than roots or rootstocks (Lubbe et al., 2023). All plant structures were placed into separate labelled paper bags, dried at 65°C for at least 48 hours and weighed. From these, we calculated dry weight (g, DW) of each plant part per pot. These data were then used for the subsequent analyses.

### 2.3 Data analyses

To answer the first two questions, we ran split-plot ANOVAs to test the effect of nutrient addition and water level on maximum plant height, plant modular dry weight (DW) (leaves, stems, roots, rhizomes and rootstocks) as well as the respective biomass allocation ratios (plant structure DW/total plant DW) and shoot number, following natural logarithmic or square root transformations if needed. These analyses were conducted in R v 4.4 (“Puppy Love”) using the nlme (Pinheiro et al., 2016), car (Fox and Weisberg, 2023) and emmeans (Lenth, 2024) packages. Nutrient, water level, species and time period (biweekly for shoot number and plant height; month of harvest for the DW measures) were the fixed effects while basin was considered as a random factor. In all cases, there was never a significant basin effect thus only the results of the linear models are shown.

In addition, changes in the mean DW of each plant module across the nutrient and water level treatments were determined as mean reaction norms. From these, we calculated whether these reaction norms followed linear or non-linear (power equation) trajectories (Gomulkiewicz and Stinchcombe, 2022). Since there were only two nutrient level treatments, these automatically followed a linear path. Thus, any analysis incorporating non-linear equations could only be conducted on the water level treatment data. These analyses were run in R v. 4.4 using the nlme package (Pinheiro et al., 2016).

Changes in plant biomass allocation patterns may be actual responses to different environmental conditions (real phenotypic plasticity) but may also only be related to changes in plant size (allometric relationship = apparent phenotypic plasticity; Weiner, 2004). We followed the procedure outlined by Jameson et al. (2022) to determine whether biomass allocation was related to plant size. First, we performed the traditional size-independent analysis determining the effect of the treatment factors on the biomass allocation ratios. For this, we conducted the aforementioned split-plot analyses on leaf, stem and root weight ratios (LWR, SWR, RWR, respectively). Then we conducted size-dependent analyses, for which the DW of the particular plant structure was divided by the total DW of the plant minus the DW of that structure, for example, LWR = leaf DW/(total plant DW- leaf DW) since using total plant DW would result in loss of independence between the tested factors (Jameson et al., 2022). For this analysis, we compared both methods of calculating the biomass allocation ratios.

Size-dependent allocation relationships were analyzed by fitting three possible allometric equations to the data relating plant structure DW to plant size based on graphical inspection of this relationship. The three models used were:

1) an isometric equation (R=cV, where R = plant structure DW, V = total plant DW excluding the DW of the particular plant structure, c = a scaling factor).

A good fitting isometric model would indicate that the allometric relationship is not affected by changing environmental conditions. In addition, we used two non-linear allometric models:

2) a power equation (R=cV^α^, where α is an allocation coefficient that is influenced by the treatment conditions) and
3) a hump equation (R=cV^αV^) (Oddi et al., 2019; Jameson et al., 2022).

For comparison, we also included a non-allometric, null model (R=c; Equation 4) in which plant size was not included. Analyses of these models were run in R v 4.4 using the nls function in the nlme package (Pinheiro et al., 2016) following natural logarithmic or square root data transformations based upon the results of bivariate normal analyses (Legendre, 2022).

Species plasticity was determined using the mean ratio values for each nutrient and water level combination separately for the July and September harvests. Plasticity was determined by subtracting the ratio values of the unfertilized samples from those that were fertilized within each water level. A positive value would show an increase in allocation to that particular structure while a negative value would indicate decreased allocation. The percent change was calculated by dividing that difference by the unfertilized value and then multiplying by 100 (Jameson et al., 2022).

Because none of our plants flowered, we were limited to using a measure of vegetative reproduction to describe the treatment effects on plant fitness (Shipley et al., 2016). Since both species are capable of producing ramets, we used shoot number as this measure. Treatment effects on the biweekly measures of shoot number were analyzed in the same manner as for the other data (split-plot ANOVA; the size-dependent models). In the case of the size-dependent analyses, we analyzed the relationship between shoot number and total plant DW.

## 3 Results

### 3.1 Environmental effects on plant biomass and allocation ratios

**Figure 1:**
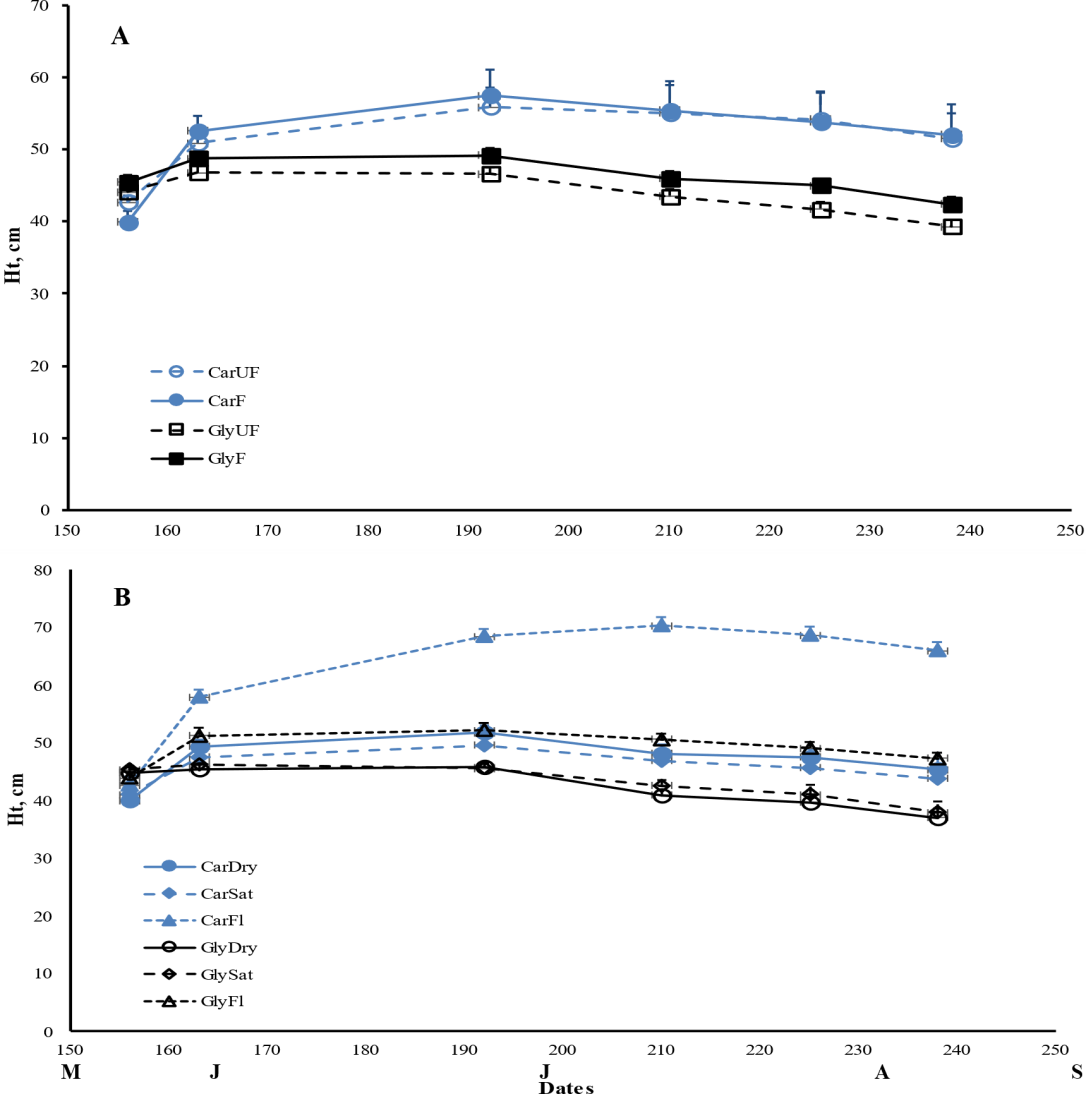
Mean maximum plant height (± 1SE) for *Carex acuta* (Car) and *Glyceria maxima* (Gly) in relation to **(A)** nutrient addition and **(B)** water level over the experimental growing period, early June to early September. Treatments: Nutrient addition – UF=unfertilized; F = fertilized (350 kg NPK ha^-1^ yr^-1^)/Water level - Dry=15 cm below the soil surface; Sat=saturated (water level at the soil surface); Fl=flooded (15 cm above the soil surface).

**Figure 2:**
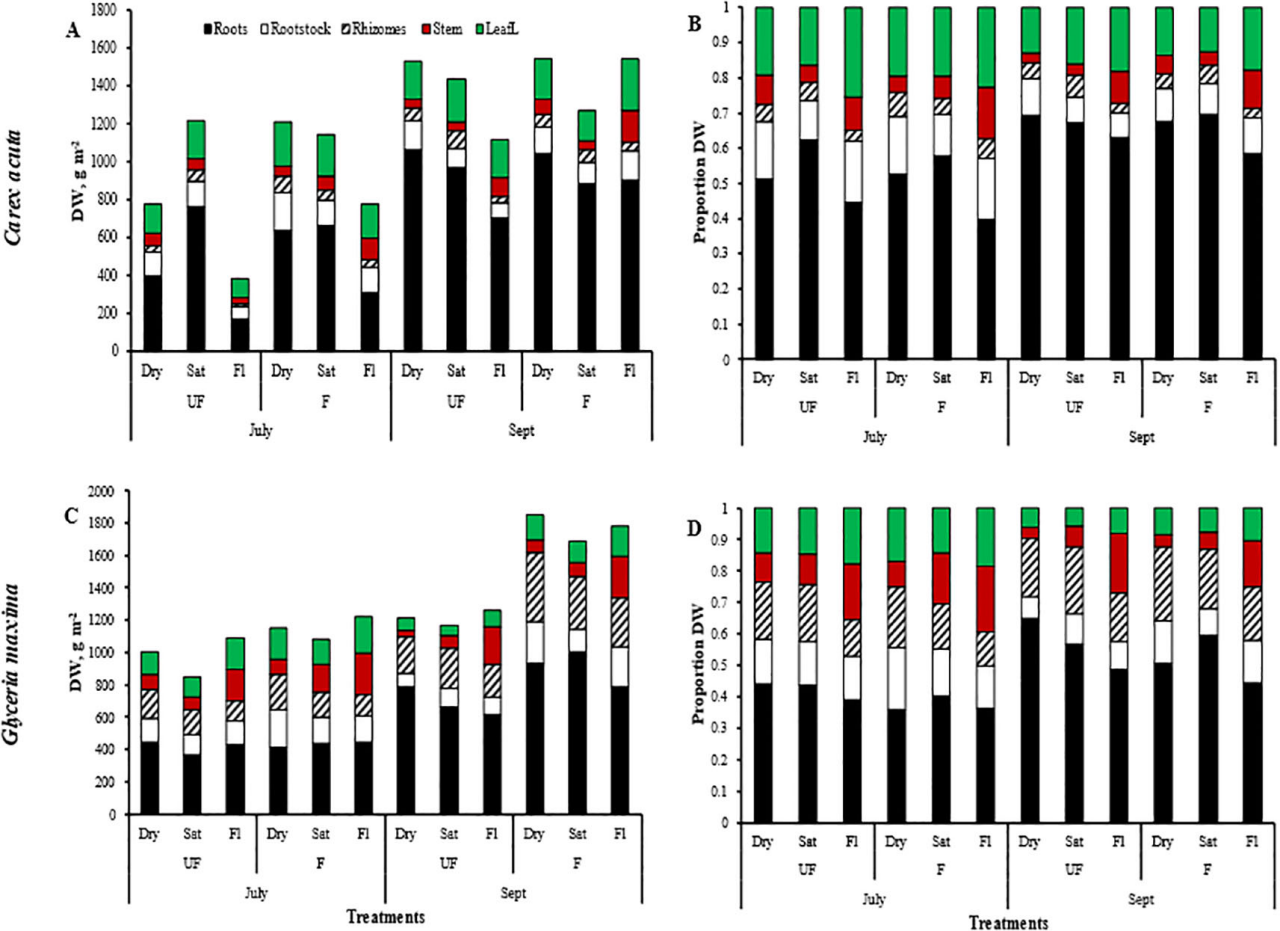
Plant module dry weights (DW; **A, C**) and DW proportions **(B, D)** for *Carex acuta* **(A, B)** and *Glyceria maxima* **(C, D)**. Treatments: Month = harvest times/Nutrient addition – UF=unfertilized; F = fertilized (350 kg NPK ha^-1^ yr^-1^)/Water level - Dry=15 cm below the soil surface; Sat=saturated (water level at the soil surface); Fl=flooded (15 cm above the soil surface).

**Figure 3:**
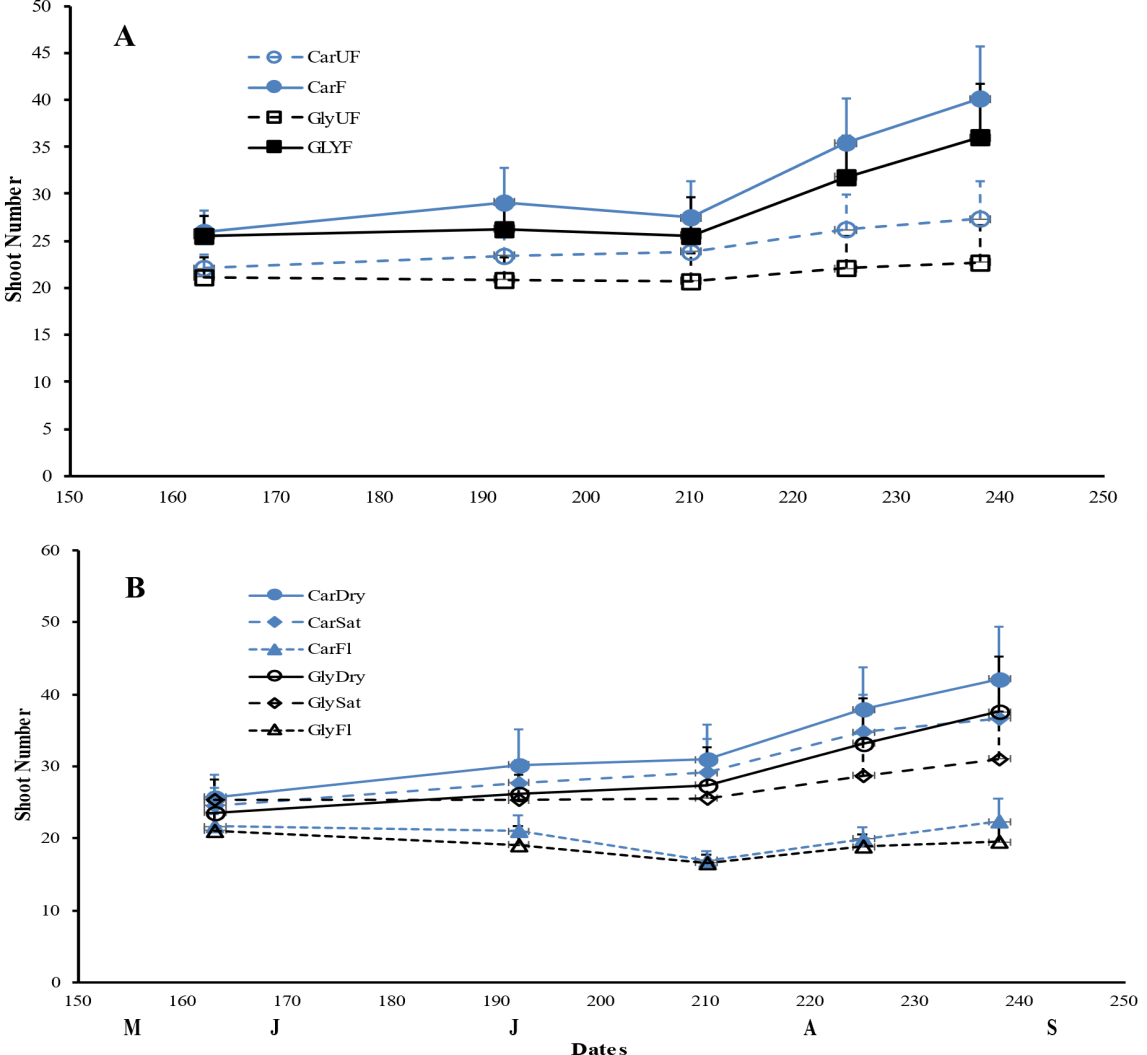
Mean shoot number (± 1SE) for *Carex acuta* (Car) and *Glyceria maxima* (Gly) in relation to **(A)** nutrient addition and **(B)** water level over the experimental growing period, early June to early September. Treatments: Nutrient addition – UF=unfertilized; F = fertilized (350 kg NPK ha^-1^ yr^-1^)/Water level - Dry=15 cm below the soil surface; Sat=saturated (water level at the soil surface); Fl=flooded (15 cm above the soil surface).

As expected, plant height and total biomass increased throughout the growing period, with maximum plant height occurring in late July, followed by a slight decrease to the end of the experiment. Biomass, especially root DW and total belowground biomass, reached their peak in September compared to July (**Figures 1**, **2**). Ramet production, our measure of vegetative reproduction, was greatest at the end of the experiment with *C. acuta* producing more new shoots than *G. maxima* (**Figure 3**). There was a significant basin effect (p < 0.001) with this spatial effect being greater earlier in the growing season but disappearing by the time of the two harvests. The production of new shoots had a linear relationship over time for both species but showed a significant increase for *C. acuta* while *G. maxima* had a more constant number of ramets except when fertilized or under dry conditions (**Figure 3**).

**Figure 4:**
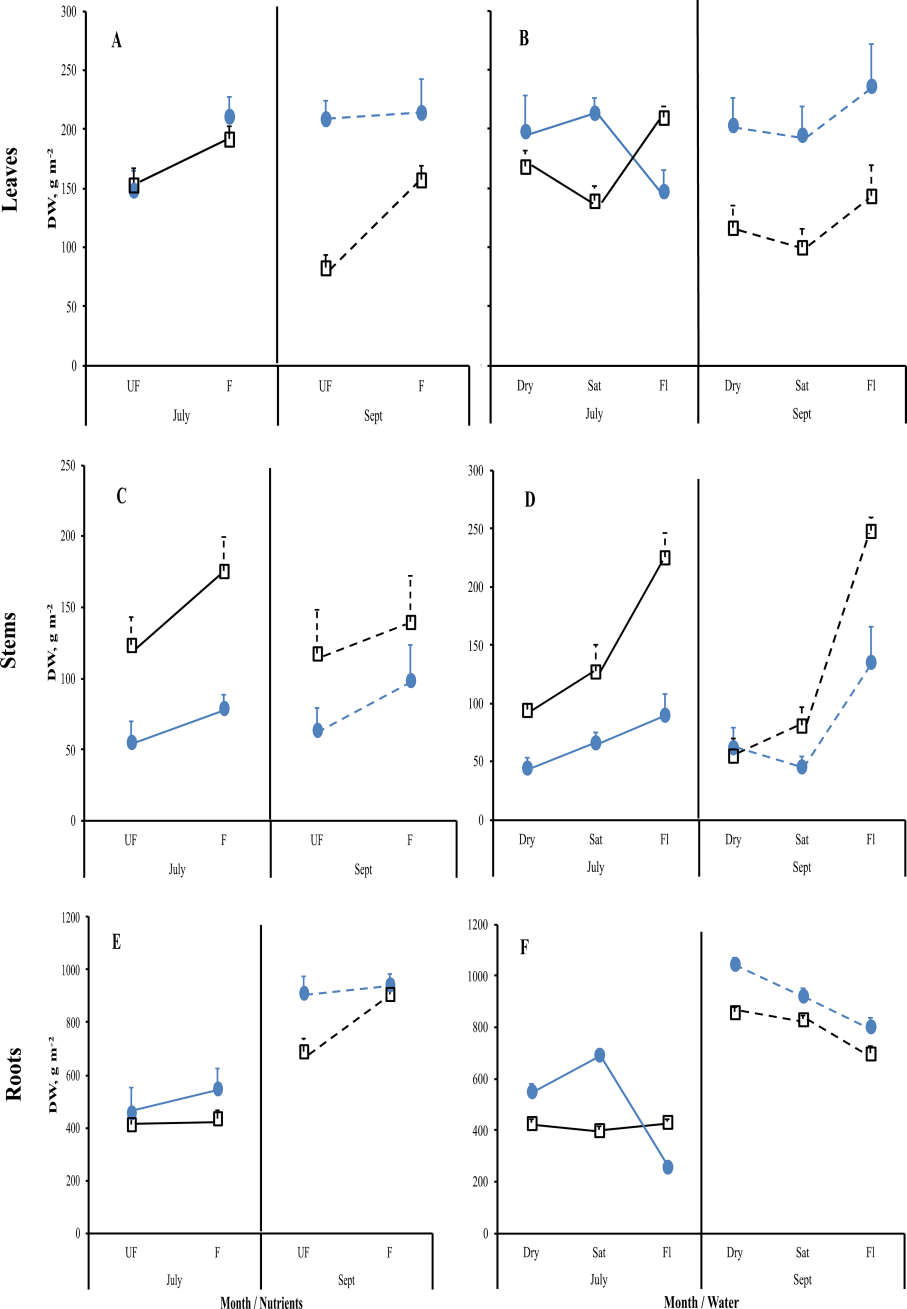
Mean reaction norms (± 1SE) of leaf **(A, B)**, stem **(C, D)**, and root **(E, F)** dry weights for *Carex acuta* (blue circles) and *Glyceria maxima* (open black squares) in relation to nutrient addition **(A, C, E)** and water level **(B, D, F)** for the July (solid lines) and September (Sept; dashed lines) harvests. Treatments: Nutrient addition – UF=unfertilized; F = fertilized (350 kg NPK ha^-1^ yr^-1^)/Water level - Dry=15 cm below the soil surface; Sat=saturated (water level at the soil surface); Fl=flooded (15 cm above the soil surface).

**Figure 5:**
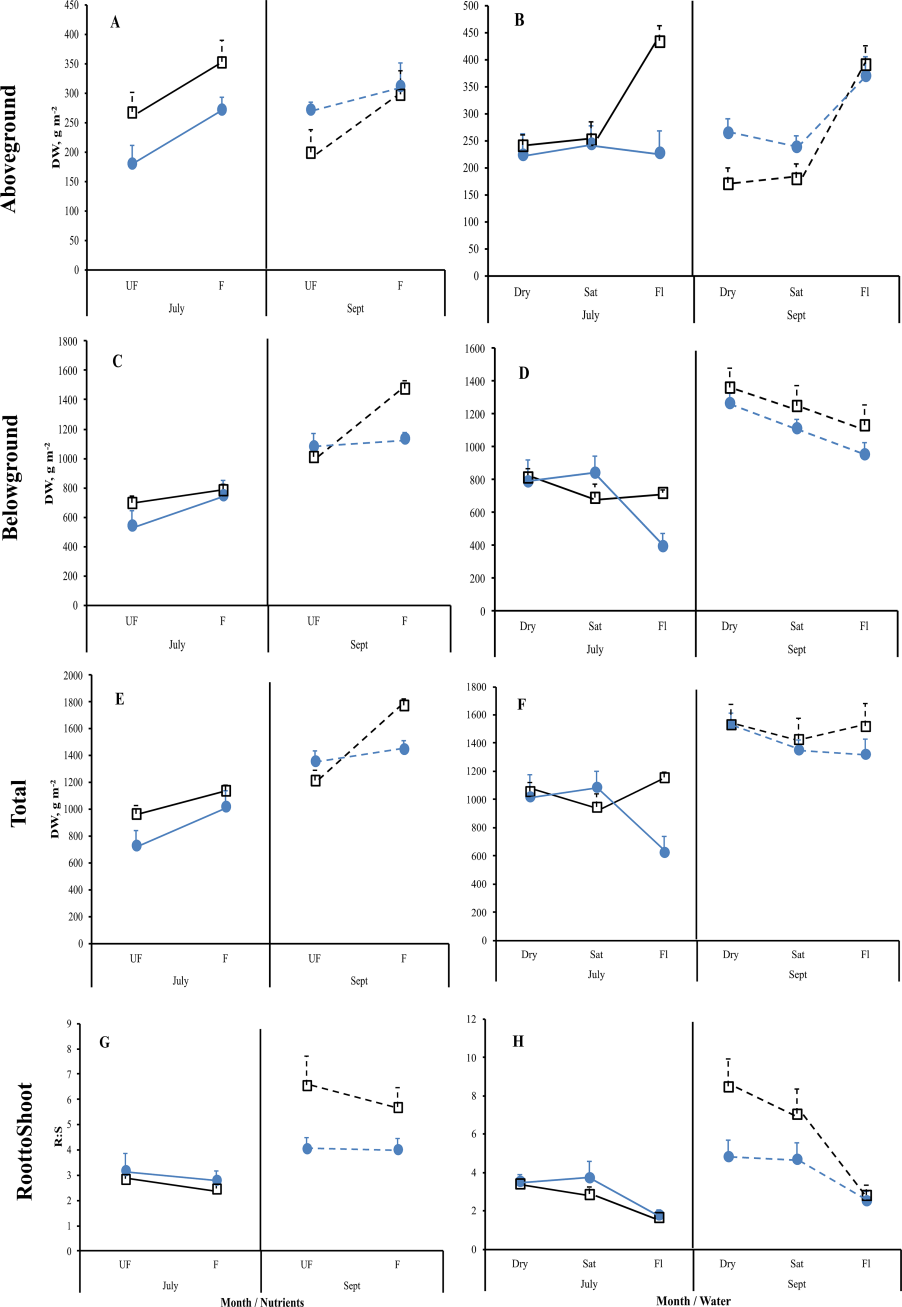
Mean reaction norms (± 1SE) of aboveground **(A, B)**, belowground **(C, D)** and total plant **(E, F)** dry weights (DW), and the root-to-shoot (R:S) ratio **(G, H)** for *Carex acuta* (blue circles) and *Glyceria maxima* (open black squares) in relation to nutrient addition **(A, C, E, G)** and water level **(B, D, F, H)** for the July (solid lines) and September (Sept; dashed lines) harvests. Treatments: Nutrient addition – UF=unfertilized; F = fertilized (350 kg NPK ha^-1^ yr^-1^)/Water level - Dry=15 cm below the soil surface; Sat=saturated (water level at the soil surface); Fl=flooded (15 cm above the soil surface).

Nutrient addition resulted in taller plants (**Figure 1**) while, except for roots, the mass of all plant parts were significantly greater with fertilization (**Figure 2**; **Table 1**). This positive nutrient effect was seen for both species, which is counter to our first prediction, but it also depended on the particular plant part. *G. maxima* had greater stem biomass, both in absolute (stem DW) and relative (SWR) terms in agreement with our first hypothesis. However, *C. acuta* had significantly more leaf DW and a greater allocation to leaves (LWR), especially in September (**Figure 4B**). In fact, *C. acuta* live leaf mass did not differ between July and September while there was a large decrease for *G. maxima* by September, which coincided with a significant increase in dead leaf DW for the latter species (**Table 1**), indicating that leaf senescence in *G. maxima* was greater and started earlier than in *C. acuta*. Also, in keeping with our first hypothesis, live leaf DW for *G. maxima* was greater in fertilized conditions in September while there was no such nutrient effect on *C. acuta* (month * species * nutrient interaction, p < 0.01). Because of these between-species differences in leaf and stem biomass, the species had similar total live aboveground biomass (**Table 1**). Also, due to greater rhizome and rootstock masses, *G. maxima* plants allocated more biomass belowground (higher live belowground DW and R:S ratio), also opposite to what we predicted (hypothesis 1), even though *C. acuta* allocated more biomass to roots (**Table 1**; **Figures 4**, **5**). Overall, there was little evidence that fertilization favored *G. maxima*, which would be noted as a significant species * fertilization interaction and which was found only for rhizomes.

**Table 1:** Results of split-plot ANOVAs (F statistic) determining the effect of the experimental treatments on (A) plant module dry weight (DW) and (B) shoot number and plant height.

A										
Parameter/Factor	Month	Species	Fert	Water	M*S	M*F	M*W	S*F	S*W	F*W
Leaf DW – live	3.92 * J > S	17.50 *** Car > Gly	12.85 *** F > UF	4.58 + F > D > S	18.83 ***				9.42 **	
Leaf DW - dead	239.87 *** S > J	227.73 *** Gly > Car	6.72 * F > UF		6.11 *	4.08 *				
Stem DW		41.39 *** Gly > Car	13.49 *** F > UF	101.20 *** F > S > D	4.61 *		8.92 *		11.32 **	
Root DW	249.53 *** S > J	3.33 + Car > Gly		40.96 *** D > S > F			5.27 +		22.48 ***	
Rootstock DW		4.61 * Gly > Car	14.66 *** F > UF							
Rhizomes DW	34.64 *** S > J	391.82 *** Gly > Car	6.12 * F > UF	10.23 *** D > S > F	15.77 ***			5.37 *	3.52 *	
Above DW	4.95 * J > S		42.84 *** F > UF	50.65 *** F > S > D	26.35 ***		7.82 **	3.13 +	17.85 ***	
Below DW	196.59 *** S > J	17.76 *** Gly > Car	23.44 *** F > UF	19.86 *** D > S > F					7.46 **	2.65 +
R:S	112.00 *** S > J	3.51 + Gly > Car	2.58 + UF > F	172.56 *** D > S > F	18.99 ***					

B										
Parameter/Factor	Time	Species	Fert	Water	T*S	T*F	T*W	S*F	S*W	F*W
Shoot Density	43.01 ***	135.18 *** Car > Gly	27.03 ** F > UF	141.51 *** D > S > F	10.37 **	33.93 ***	49.01 ***		21.99 ***	12.59 ***
Height	53.16 ***	47.70 *** Car > Gly	7.95 *** F > UF	180.61 *** F > S > D	109.95 ***		67.11 ***		7.80 ***	

Only significant effects shown. Treatments: Month (A): July (J); September (S). Species: *Carex acuta* (Car); *Glyceria maxima* (Gly). Fert: nutrient addition = unfertilized (UF); fertilized (F = 350 kg NPK ha^-1^ yr^-1^). Water: water level = dry (D = 15 cm below the soil surface); saturated (S = at the soil surface); flooded (Fl = 15 cm above the soil surface). Time (B): biweekly intervals. R:S, root-to-shoot ratio; P values: + < 0.10; * < 0.05; ** < 0.01; *** < 0.001.

While leaf DW was affected more by fertilization (**Table 1**; **Figures 4A, B**), water level had a greater impact on both stem and root DW (**Figures 4E, F**). Of the three water level treatments, flooding appeared to be the more stressful condition, while results were similar for the dry and saturated treatments. Flooding led to greater stem growth, but significantly diminished ramet production in *G. maxima* (species × water interaction, p < 0.001; **Figures 4C, D**) while it resulted in decreased root mass in *C. acuta* (**Figures 4E, F**). There were fewer new shoots when no additional nutrients were applied (nutrient × water interaction, p < 0.001) as predicted (hypothesis 2). As a result, more biomass was allocated aboveground when the plants were flooded, especially in September (**Figure 5H**). Also, there was little evidence to support our contention that hydrology would alter any nutrient effect on biomass and the allocation pattern (second hypothesis) with only rhizome DW and RWR having significant nutrient * water interactions (**Table 1**).

### 3.2 Allometric relationships

Both species showed positive plastic responses to fertilization, with the exceptions of root DW in July and *C. acuta* leaf and root DWs in September (**Figure 4**), and to changing hydrology with *C. acuta* having greater plasticity in belowground structures (**Figure 5D**), and possibly leaf DW, while there was larger plasticity in aboveground structures shown by *G. maxima* (**Figure 5B**). Further analyses were then conducted to determine whether this plasticity was only due to changes in plant size.

#### 3.2.1 Size independent analyses

Overall, the allocation ratios and non-linear power models gave the best results, based on AIC, while the null and isometric models never fit well with the data (**Supplementary Table S1**). All of the experimental factors (month, species, water level, nutrient addition) significantly affected the biomass allocation ratios, usually singly although there were important month × species interactions especially with LWR and RWR for *C. acuta* plants in July and September respectively, while *G. maxima* SWR was much greater in July compared to the other combinations (**Table 2**). For both species, nutrient addition tended to positively affect LWR and SWR plasticity but had a negative impact on RWR plasticity.

**Table 2:** Results of size-independent split-plot ANOVAs (F values) determining the effect of the experimental treatments on the biomass allocation ratios.

Parameter/Factor	Month	Species	Fert	Water	M*S	M*W	S*W	S*F	F*W	AIC
LWR	147.01 *** J > S	134.61 *** Car > Gly	5.31 * F > UF	19.17 *** F > S > D	16.70 ***					-444.64
SWR	49.12 *** J > S	19.37 *** Gly > Car	4.34 * F > UF	72.92 *** F > S > D	4.03 *					-308.18
RWR	296.42 *** S > J	189.52 *** Car > Gly	16.62 *** UF > F	49.84 *** S > D > F				8.23**	4.40*	-475.78
StockWR	47.35 *** J > S		6.95 ** F > UF	2.87 + D > F > S						181.66
RhizWR		403.74 *** Gly > Car		10.35 *** D > S > F	5.16*					-253.68

Only significant effects shown. Treatments: Month (M): July (J); September (S). Species (S): *Carex acuta* (Car); *Glyceria maxima* (Gly). Fert (F): nutrient addition = unfertilized (UF); fertilized (F = 350 kg NPK ha^-1^ yr^-1^). Water (W): water level = dry (D = 15 cm below the soil surface); saturated (S = at the soil surface); flooded (Fl = 15 cm above the soil surface). LWR, leaf weight ratio; SWR, stem weight ratio; RWR, root weight ratio; StockWR, rootstock weight ratio; RhizWR, rhizome weight ratio; AIC, Akaike information criteria. P values: + < 0.10; * < 0.05; ** < 0.01; *** < 0.001.

Both species showed high plasticity for the size-independent analyses, but this depended on season as well as plant part. Both species had similar leaf plasticity in July, with leaf DW increasing with fertilization, with maximum leaf plasticity occurring in the saturated treatment for *C. acuta* but dry conditions for *G. maxima* (**Table 3**). Fertilization had a negative impact on leaf DW for *C. acuta* in September while it still had a positive effect on *G. maxima*. Overall, *G. maxima* was the more plastic species for stem and root DW, consistent with our prediction (hypothesis 4). The plasticity of vegetative reproduction was also positively impacted by nutrient addition in all water level treatments with the exception of flooded *G. maxima* plants in July (**Table 3**).

**Table 3:** Plasticity in allocation of plant modular structures measured as the difference in mean allocation ratios (Ratio) for *Carex acuta* (Car) and *Glyceria maxima* (Gly).

Ratio	Month	Species	Water	F-UF	% Change
LWR	July	Car	Dry	0.021	9.06
			Sat	0.055	26.61
			Flood	0.006	1.79
		Gly	Dry	0.038	23.48
			Sat	0.028	17.40
			Flood	0.012	5.76
	September	Car	Dry	0.009	5.57
			Sat	-0.044	-22.80
			Flood	-0.002	-0.79
		Gly	Dry	0.027	40.38
			Sat	0.024	38.30
			Flood	0.023	24.92
SWR	July	Car	Dry	-0.004	-7.78
			Sat	0.017	31.07
			Flood	0.035	19.58
		Gly	Dry	-0.005	-5.07
			Sat	0.082	88.64
			Flood	0.052	23.98
	September	Car	Dry	0.023	68.89
			Sat	0.009	29.27
			Flood	0.021	20.24
		Gly	Dry	0.006	18.32
			Sat	-0.002	-2.81
			Flood	-0.028	-14.32
RWR	July	Car	Dry	-0.130	-10.36
			Sat	-0.259	-16.21
			Flood	-0.200	-23.36
		Gly	Dry	-0.275	-31.84
			Sat	-0.305	-34.61
			Flood	-0.087	-13.22
	September	Car	Dry	-0.266	-11.02
			Sat	0.206	9.63
			Flood	-0.307	-17.60
		Gly	Dry	-0.880	-45.57
			Sat	0.056	3.92
			Flood	-0.174	-17.69
Shoot No	July	Car	Dry	12.417	34.98
			Sat	1.000	3.18
			Flood	6.583	27.53
		Gly	Dry	6.500	36.79
			Sat	4.917	28.37
			Flood	-0.083	-0.52
	September	Car	Dry	25.083	64.18
			Sat	10.667	26.28
			Flood	9.667	46.40
		Gly	Dry	13.333	62.26
			Sat	12.583	81.18
			Flood	6.667	51.95

Differences between the two nutrient treatments (UF = unfertilized; F = fertilized (350 NPK kg ha^-1^ yr^-1^) calculated for month (July, September), species and water levels (Dry = 15 cm below the soil surface; Sat = saturated (water level at the soil surface); flood = 15 cm above the soil surface). LWR, leaf weight ratio; SWR, stem weight ratio; RWR, root weight ratio; Shoot No, shoot density.

#### 3.2.2 Size-dependent analyses

Similarly as with the size independent analyses, leaf, stem and root masses were significantly affected by all of the treatment factors (**Table 4**). The main difference was that there were more treatment interactions in the size dependent analyses, notably species × water interactions. For example, *C. acuta* leaf and root DWs, as well as stem number, were greater than those of *G. maxima* especially in dry and saturated conditions. On the contrary, *G. maxima* had the highest stem DW when flooded while that of *C. acuta* was the lowest in dry and saturated conditions. In addition, the water level effect on stem and root DWs differed between July and September (season × water interaction) with stem DW being the lowest under dry conditions in September while it was greater when flooded in both months. Likewise, root DW was the highest in September, especially in dry conditions, but the lowest when flooded in July.

**Table 4:** Comparison of three size dependent (1: isometric; 2: power; 3: hump) equations and a size-independent null model (4) for shoot number as a measure of vegetative reproduction.

Water	Equations	July				September			
		UF		F		UF		F	
		Carex	Glyceria	Carex	Glyceria	Carex	Glyceria	Carex	Glyceria
Dry	1) R=cV	7.67 (6.15)	2.40 (0.42)	6.14 (5.30)	3.51	15.46	1.18 (0)	0	6.82
	2) R=cV^α^	1.52 (0)	1.98 (0)	0.85 (0)	0.52	0	1.92 (0.74)	1.44	0
	3) R=cVe^αV^	3.43 (1.91)	2.23 (0.25)	1.41 (0.57)	0	17.44	1.90 (0.71)	1.44	NA
	4) R=c	0	0	0	0.77	16.84	0	1.31	4.75
Sat	1) R=cV	5.18	0.78	2.15	2.47 (1.55)	10.89	21.63	6.26 (4.40)	2.69
	2) R=cV^α^	0	0	0.01	1.02 (0.10)	0.79	4.85	1.98 (0.13)	0
	3) R=cVe^αV^	2.71	0.48	0	0.92 (0)	0	0	1.86 (0)	0.16
	4) R=c	3.77	4.58	1.47	0	18.32	9.50	0	0.17
Flood	1) R=cV	0.61	3.07 (1.22)	5.32 (3.91)	0	3.24 (1.60)	4.70 (3.86)	0	4.22 (2.47)
	2) R=cV^α^	0	1.84 (0)	1.42 (0)	1.95	1.64 (0)	0.84 (0)	1.60	1.75 (0)
	3) R=cVe^αV^	0.52	1.79 (0.05)	2.48 (1.07)	1.95	1.89 (0.24)	20.3 (1.19)	1.61	2.02 (0.27)
	4) R=c	1.15	0	0	5.68	0	0	3.03	0

Differences in Akaike information criteria (AIC) values are shown. Equations with a zero value had the lowest AIC score. Values in parentheses represent AIC differences when comparing the three size-dependent models only. Comparisons > 2 are significantly different. Equations: R = shoot number; V = plant dry weight; c, α = scaling factors. Nutrient addition: UF = unfertilized; F = fertilized (350 NPK kg ha^-1^ yr^-1^)/Water level: Dry = 15 cm below the soil surface; Sat = saturated (water level at the soil surface); flood = 15 cm above the soil surface. NA = samples which did not converge with the model. Based on Oddi et al. (2019) and Jameson et al. (2022).

Contrary to our third hypothesis, we found no consistent indication for allometric effects in any of the measured parameters (**Supplementary Tables S2-S4**). Stem DW and number had the fewest such relationships especially when flooded (**Table 4**; **Supplementary Table S3**). Leaf DW also showed no allometric relationships in September for both species, notably when saturated or flooded (season × water interaction; **Supplementary Table S2**). However, allometric relationships were most common for root DW (**Supplementary Table S4**), especially for *C. acuta* in July, in which all three size-dependent models could sufficiently represent the results, while this was the case for *G. maxima* only in July under dry conditions. This differed in September, when the power and hump models (Equations 2, 3; **Supplementary Table S4**) could adequately express the root DW to total plant weight minus root weight relationship.

## 4 Discussion

The aim of our study was to describe the niches of two common wet grassland species, *C. acuta* and *G. maxima*, by investigating changes in their life history characteristics as a result of changing environmental conditions (fertilization, water level). Determining how the species respond to these environmental changes, and where and by how much their respective niches may overlap, would help us to understand how these two species can sometimes co-exist in wet grasslands. Our initial hypotheses could be divided into two groups, the first dealing with nutrient and water level effects on plant growth and biomass allocation patterns (hypotheses 1, 2) while the second group of hypotheses were more concerned with whether the observed phenotypic plasticity followed an allometric relationship (hypotheses 3, 4). While some of our results supported our hypotheses, many other results were quite the opposite of what was expected.

### 4.1 Environmental effects greatly impact plant traits

According to the “fast-slow” plant economic spectrum (Reich, 2014), more competitive plant species should dominate in higher resource conditions, having faster growth and more biomass allocated to aboveground structures, while stress tolerators should have traits indicative of the conservative plant strategy, namely slower growth with greater biomass allocation to belowground structures (De Deyn, 2017). At the onset of our study, we considered *G. maxima* to be the more competitive species since it is often found in nutrient-richer habitats. The high nutrient contents found in its plant structures indicate a high uptake of available nutrients, which make it a valuable fodder plant (Lamber, 1947; Mugwedi et al., 2014). These traits have also resulted in *G. maxima* becoming a very invasive species not only in areas outside of its native range in temperate Europe and Asia (North America, Australia, New Zealand, South Africa) but even in more northern locations in Europe (Ireland and Scandinavia; Mugwedi et al., 2014; NOBANIS, 2018). Therefore, we expected that *G. maxima* would act more like a competitive species, having greater growth in fertilized conditions with more biomass allocated to aboveground structures, with a concomitant lower R:S ratio, than *C. acuta*, which should have features characteristic of stress-tolerators (Menges and Waller, 1983; Edwards and Čížková, 2020). Our results only partially supported our first hypothesis. As predicted, the *C. acuta* plants in our study had greater root DW than *G. maxima*, while the latter species had greater aboveground biomass when fertilized. However, contrary to the first hypothesis, total live belowground DW was greater in *G. maxima*, due to this species having significantly larger rootstock and rhizome masses. This resulted in *G. maxima* having larger R:S ratios, notably in unfertilized conditions (**Table 1**). Nutrient addition did result in greater allocation of biomass to aboveground structures in *G. maxima* compared to unfertilized plants (**Table 1**), while there was no nutrient effect on the R:S ratio of *C. acuta*. This greater impact of fertilization on *G. maxima* is in agreement with the first hypothesis. Overall, however, these biomass allocation results only partially support our first hypothesis.

Also contrary to expectations, *C. acuta* produced a greater number of new ramets, having a more compact growth form than *G. maxima*, which produced fewer but more spatially separated ramets, by which it could grow into bare areas within or between *C. acuta* clumps (Glocker et al., 2024). Since shoot density is a proxy measure of plant fitness (Shipley et al., 2016), the greater number of ramets would indicate that *C. acuta* is more fit especially in dry and saturated conditions. In addition, the greater production of ramets by *C. acuta* later in the growing season is likely the reason why live leaf DW did not differ between July and September for this species, even though the amount of dead leaf DW did increase near the end of the growing season. Thus, *G. maxima* starts to die back and senesce earlier than *C. acuta*. Both species were stressed when flooded, with *C. acuta* and *G. maxima* responding to prolonged flooding by increasing their allocation to stem and leaf growth, which is a well-known response of wetland plants (Pezeshki, 2001). However, counter to expectations, this increase in shoot height was much greater for *C. acuta* than that of the supposedly more competitive species. Plant height has often been linked to competitive ability (Tilman, 1982; Givnish, 1982), thus, by that metric, *C. acuta* should be considered as the better competitor than *G. maxima* if only above ground structures are considered. However, a different picture emerges when belowground structures are included. The R:S ratio decreased in both harvests for both species when flooded (**Figure 5H**), but this reduction was due to different growth patterns in the two species, especially at the peak time of the growing season in July. At that time, leaf and stem growth increased for *C. acuta*, but the overall mass of the aboveground structures remained the same. This stability is likely associated with the smaller number of ramets produced by *C. acuta* when flooded so that, on an area basis, there were larger but fewer leaves, which led to the stable aboveground biomass unaffected by water level changes. Concurrently, root growth decreased in *C. acuta* which, associated with the lack of change in aboveground DW, resulted in the decreased R:S ratio. *G. maxima* showed the opposite trends at the same point of the growing season, with this species allocating more biomass to above ground structures while allocation to root mass was unaffected by flooding. Also, while the new ramet production by *G. maxima* was reduced when flooded, it was not as affected by changing water level conditions as *C. acuta*. By September, both species responded in a similar manner, with both allocating more mass to above ground and less to below ground structures. These results indicate that *C. acuta* will be more stressed under prolonged flooding thereby likely reducing its competitive ability. Unfortunately, direct competition between the two species was not measured in our study so we cannot categorically state whether *C. acuta* may be able to outcompete *G. maxima* under the experimental conditions used here.

Two or more plant species may co-exist if they inhabit different niches (Silvertown, 2004). In turn, biomass allocation patterns are influenced externally by these environmental factors and internally by the life history strategy of the particular species (Metcalf et al., 2006; De Deyn, 2017). While there are areas of overlap in the niches of the two species (similar total above and belowground live DW), there are also important differences (allocation ratios, stem number, height) which would make co-existence more likely under certain environmental conditions (Chesson, 2000). Future studies incorporating direct species interactions are needed to test this idea.

Our results indicate that *G. maxima* should dominate in nutrient-rich wet grasslands and at times of prolonged flooding and in slight depressions, while *C. acuta* would be favored at times of lower water levels regardless of the nutrient condition of the site, which is, in fact, the zonation observed in the field (Hroudová et al., 1988; Edwards et al., 2024). This prediction differs from that of Edwards and Čížková (2020), who predicted that, while *G. maxima* should dominate in nutrient-richer conditions as in our study, flooding would favor *C. acuta*. This discrepancy in the results of the two studies may be due to the fact that Edwards and Čížková (2020) only considered net annual aboveground production of these two species in years differing in hydrologic conditions, but did not incorporate belowground biomass as we did here. As stated above, even in our study, flooding led to greater leaf and shoot growth in *C. acuta*, which likely led to the greater above ground production noted by Edwards and Čížková (2020). Therefore, our results emphasize the importance of including belowground plant structures in such studies (also see De Kroon et al., 2012; Bardgett et al., 2014).

### 4.2 Possible allometric relationships

Phenotypic plasticity is the ability of a plant to produce different phenotypes under changing environmental conditions (Pigliucci, 2001), with plant modular units being the level at which plasticity is expressed and analyzed (De Kroon et al., 2005). Biomass allocation ratios and reaction norms are common methods for analyzing phenotypic plasticity. Here, both species showed plastic responses to changing nutrient and water level conditions, which was opposite of what we initially predicted (hypothesis 4), with these changes having a seasonal aspect. However, while some of these changes were also related to plant size (Schneider, 2022; Gomulkiewicz and Stinchcombe, 2022), our overall results were inconsistent with other responses having no allometric relationship. Therefore, our third hypothesis that most of the plastic responses would be attributed to changes in plant size cannot be supported.

Counter to our prediction, in only a few cases there was a clear relationship between the production of new ramets and plant size, with the fewest occurring when the plants were flooded. Most such relationships were found for *C. acuta* (**Table 4**). This is in contrast to Jameson et al. (2022) who found a larger number of size-dependent relationships. However, these authors used sexual reproduction as their measure of fitness. Given that none of our plants flowered, we had to use vegetative spread as our measure of fitness. Therefore, it is not known whether sexual reproduction would show such allometric relationships.

A similar lack of an allometric relationship was noted in the size-dependent model analyses for leaves and stems but such relationships were more common with roots. Again, *C. acuta* had more size-dependent relationships than *G. maxima*. In addition, time of the growing season determined whether size-dependent relationships were possible. For example, the response of leaf DW to flooding did show a clear allometric relationship in July for both species, but this had largely disappeared by September, notably for *C. acuta*, likely reflecting plant development patterns in that the plants had begun to senesce by early September (Schneider, 2022). However, there was no such seasonal effect for *G. maxima* (**Supplementary Table S1**). In fact, while leaf DW decreased from July to September, nutrient addition maintained these structures, likely prolonging the *C. acuta* growing season.

## 5 Conclusions

In our study, *C. acuta* and *G. maxima* did not behave as expected according to the general predictions about conservative and competitive species. We found that:

1. as predicted, *G. maxima* was more affected by fertilization than *C. acuta*. However, counter to our prediction, *G. maxima* allocated more biomass to below ground than above ground structures having higher R:S ratios than *C. acuta*. Therefore, our results emphasize the importance of including belowground plant structures in such studies;
2. counter to our second hypothesis, changes in site hydrology affected *C. acuta* more than *G. maxima*, especially regarding stem DW, plant height and ramet production. In addition, water level had a stronger effect on the R:S ratio than fertilization in both species with below ground biomass decreasing for both species when flooded as compared to drier conditions;
3. both species showed plastic responses to changing environmental conditions but only some were related to plant size. *C. acuta* produced more daughter shoots than *G. maxima*. Thus, we could only partially support our last two predictions that phenotypic plasticity followed an allometric relationship (hypotheses 3) and *G. maxima* was the more plastic species (hypothesis 4);
4. *C. acuta* would likely be favored in drier, lower nutrient habitats while *G. maxima* would prefer growing in wetter, nutrient-rich sites. With warmer and drier conditions predicted for central Europe because of climate change (IPCC, 2023), such a future would favor *C. acuta* to the detriment of *G. maxima*.

This information may be important for predicting possible sites that would be prone to *G. maxima* invasion as well as developing management and control plans to combat the spread and establishment of invasive populations of this species (Mugwedi et al., 2014). Overall, the allocation patterns and vegetative reproduction of the two species were often different than expected, with both species having characteristics of both the conservative and competitive plant functional types (De Deyn, 2017). Our results imply that *C. acuta* and *G. maxima* may be able to co-exist in oligo- to mesotrophic wet grasslands with fluctuating water levels as well as more heterogeneous habitats. Further studies including direct species interactions would be needed to determine the validity of this conjecture. Still, our study provides important information which would be useful for predicting and modelling the effects of management and climate change on wet grassland plant species composition as well as maintaining the ability of wet grasslands to provide their important ecosystem services.

## Data Availability

The raw data supporting the conclusions of this article will be made available by the authors, without undue reservation.
