# Supplementary material for: Changing environmental conditions impact the phenotypic plasticity of Carex acuta and Glyceria maxima, two common wet grassland species
Source: Front Plant Sci. 2025 Apr 28;16:1542907. doi: 10.3389/fpls.2025.1542907 (PMC12066794; doi:10.3389/fpls.2025.1542907)
Supplement: Supplementary file 1 [file Table1.docx]

**Supplementary table 1. Comparison of Akaike information criteria (AIC) values for four models (1: null; 2: size-independent; 3: isometric; 4: non-linear power) equations in explaining the effect of the nutrient and water level experimental treatments on modular biomass. The isometric and power models are allometric, size-dependent equations. Equations: R = shoot number; V = plant dry weight; c, α = scaling factors. Acronyms: Nutrient addition: UF = unfertilized; F = fertilized (350 NPK kg ha^-1^ yr^-1^) / Water level: Dry = 15 cm below the soil surface; Sat = saturated (water level at the soil surface); flood = 15 cm above the soil surface. NA = samples which did not converge with the model. Based on Oddi et al. (2019) and Jameson et al. (2022).**

| **Plant Module** | **Null** | **Size-independent** | **Isometric** | **Power** |
| --- | --- | --- | --- | --- |
| **Leaves** | -12.81 | -319.37 | 41.18 | -130.26 |
| **Stem** | 110.53 | -224.15 | 130.66 | 12.67 |
| **Roots** | 588.56 | 98.94 | 604.21 | 405.76 |
| **Rootstock** | 209.59 | 181.52 | 208.19 | NA |
| **Rhizomes** | 183.88 | -253.68 | 170.14 | -0.81 |

**Supplementary Table 2. Comparison of three size-dependent (1: isometric; 2: power; 3: hump) equations and a size-independent null model (4) for leaves. Differences in Akaike information criteria (AIC) values are shown. Equations with a zero value had the lowest AIC score. Values in parentheses represent AIC differences when comparing the three size-dependent models only. Comparisons > 2 are significantly different. Equations: R = leaf weight; V = plant dry weight minus leaf dry weight; c, α = scaling factors. Acronyms: Nutrient addition: UF = unfertilized; F = fertilized (350 NPK kg ha^-1^ yr^-1^) / Water level: Dry = 15 cm below the soil surface; Sat = saturated (water level at the soil surface); flood = 15 cm above the soil surface. NA = samples which did not converge with the model. Based on Oddi et al. (2019) and Jameson et al. (2022).**

| **Water** | **Equations** | **July** | | | | **September** | | | | | | |
| --- | --- | --- | --- | --- | --- | --- | --- | --- | --- | --- | --- | --- |
|  |  | **UF** | | **F** | | **UF** | | | | **F** | | |
|  |  | **Carex** | **Glyceria** | **Carex** | **Glyceria** | **Carex** | | **Glyceria** | | **Carex** | | **Glyceria** |
| **Dry** | 1. R=cV 2. R=cV^α^ 3. R=cVe^αV^ 4. R=c | 14.84 | 1.03(0) | 1.08 | 4.07 | 1.48 | 0.48 (0) | | 0 | | 9.10 | |
|  |  | 0.96 | 1.42 (0.39) | 0.25 | 0.37 | 0 | 1.90 (1.42) | | 1.83 | | 0 | |
|  |  | 0 | 1.46 (0.43) | 0 | 0 | 0.54 | 1.94 (1.46) | | 1.87 | | 0.18 | |
|  |  | 13.84 | 0 | 1.20 | 7.24 | 7.62 | 0 | | 5.05 | | 4.13 | |
| **Sat** | 1. R=cV 2. R=cV^α^ 3. R=cVe^αV^ | 11.44 | 0 | 6.00 | 7.51 | 4.16 (2.38) | 0 | | 0.74 (0) | | 5.72 (4.11) | |
|  |  | 0 | 1.11 | 0 | 0.30 | 2.00 (0.22) | 1.33 | | 1.33 (0.59) | | 1.67 (0.06) | |
|  |  | 1.56 | 1.17 | 0.01 | 0 | 1.78 (0) | 1.29 | | 1.53 (0.79) | | 1.60 (0) | |
|  | 1. R=c | 3.75 | 3.82 | 1.28 | 5.81 | 0 | 3.07 | | 0 | | 0 | |
| **Flood** | 1. R=cV 2. R=cV^α^ 3. R=cVe^αV^ | 1.95 | 0.38 | 5.43 | 4.22 (2.96) | 0.62 | 4.34 (3.93) | | 4.67 (4.51) | | 0 | |
|  |  | 0 | 0 | 0 | 1.26 (0) | 0.22 | 0.51 (0.11) | | 0.46 (0.30) | | 1.18 | |
|  |  | 0.45 | 0.03 | 0.08 | 1.28 (0.02) | 0 | 0.40 (0) | | 0.16 (0) | | 1.32 | |
|  | 1. R=c | 5.19 | 6.93 | 1.43 | 0 | 0.30 | 0 | | 0 | | 0.02 | |

**Supplementary Table 3. Comparison of three size-dependent (1: isometric; 2: power; 3: hump) equations and a size-independent null model (4) for stems. Differences in Akaike information criteria (AIC) values are shown. Equations with a zero value had the lowest AIC score. Values in parentheses represent AIC differences when comparing the three size-dependent models only. Comparisons > 2 are significantly different. Equations: R = stem weight; V = plant dry weight minus stem dry weight; c, α = scaling factors. Acronyms: Nutrient addition: UF = unfertilized; F = fertilized (350 NPK kg ha^-1^ yr^-1^) / Water level: Dry = 15 cm below the soil surface; Sat = saturated (water level at the soil surface); flood = 15 cm above the soil surface. NA = samples which did not converge with the model. Based on Oddi et al. (2019) and Jameson et al. (2022).**

| **Water** | **Equations** | **July** | | | | **September** | | | | | | |
| --- | --- | --- | --- | --- | --- | --- | --- | --- | --- | --- | --- | --- |
|  |  | **UF** | | **F** | | **UF** | | | | **F** | | |
|  |  | **Carex** | **Glyceria** | **Carex** | **Glyceria** | **Carex** | | **Glyceria** | | **Carex** | | **Glyceria** |
| **Dry** | 1. R=cV 2. R=cV^α^ 3. R=cVe^αV^ 4. R=c | 5.24 (4.49) | 2.92 (1.56) | 3.73 (1.89) | 9.17 | 13.85 | 0 | | 0.23 (0) | | 11.66 | |
|  |  | 0.74 (0) | 1.36 (0) | 1.84 (0) | 1.99 | 0 | 1.47 | | 1.78 (1.55) | | 0 | |
|  |  | 1.41 (0.67) | 1.91 (0.56) | 2.56 (0.73) | 0 | 1.96 | 1.46 | | 1.87 (1.64) | | 0.21 | |
|  |  | 0 | 0 | 0 | 5.04 | 11.44 | 2.84 | | 0 | | 8.24 | |
| **Sat** | 1. R=cV 2. R=cV^α^ 3. R=cVe^αV^ | 0 | 0 | 9.77 | 23.35 | 0 | 0.29 (0) | | 4.03 (2.63) | | 11.18 | |
|  |  | 0.36 | 1.55 | 0 | 0 | 2.00 | NA | | 1.40 (0) | | 0 | |
|  |  | 0.62 | 1.41 | 0.69 | 3.49 | 1.99 | 1.28 (0.99) | | 1.62 (0.22) | | 0.07 | |
|  | 1. R=c | 1.08 | 6.98 | 0.18 | 13.10 | 0.75 | 0 | | 0 | | 4.56 | |
| **Flood** | 1. R=cV 2. R=cV^α^ 3. R=cVe^αV^ | 7.26 | 2.67 (1.16) | 17.69 (16.55) | 11.19 (10.33) | 0.79 (0) | 0 | | 1.67 (0) | | 0 | |
|  |  | 0 | 1.52 (0) | 1.14 (0) | 0.86 (0) | 1.97 (1.18) | 1.70 | | 1.93 (0.26) | | 1.20 | |
|  |  | 0.91 | 1.62 (0.11) | 5.26 (4.12) | 1.81 (0.95) | 2.22 (1.44) | 1.78 | | 1.76 (0.09) | | 1.07 | |
|  | 1. R=c | 8.35 | 0 | 0 | 0 | 0 | 0.26 | | 0 | | 3.50 | |

**Supplementary Table 4. Comparison of three size-dependent (1: isometric; 2: power; 3: hump) equations and a size-independent null model (4) for roots. Differences in Akaike information criteria (AIC) values are shown. Equations with a zero value had the lowest AIC score. Values in parentheses represent AIC differences when comparing the three size-dependent models only. Comparisons > 2 are significant differences. Equations: R = root dry weight; V = plant dry weight minus root dry weight; c, α = scaling factors. Acronyms: Nutrient addition: UF = unfertilized; F = fertilized (350 NPK kg ha^-1^ yr^-1^) / Water level: Dry = 15 cm below the soil surface; Sat = saturated (water level at the soil surface); flood = 15 cm above the soil surface. Based on Oddi et al. (2019) and Jameson et al. (2022).**

| **Water** | **Equations** | **July** | | | | **September** | | | | | | |
| --- | --- | --- | --- | --- | --- | --- | --- | --- | --- | --- | --- | --- |
|  |  | **UF** | | **F** | | **UF** | | | | **F** | | |
|  |  | **Carex** | **Glyceria** | **Carex** | **Glyceria** | **Carex** | | **Glyceria** | | **Carex** | | **Glyceria** |
| **Dry** | 1. R=cV 2. R=cV^α^ 3. R=cVe^αV^ 4. R=c | 0 | 0 | 0.23 | 4.14 (2.51) | 23.06 | 19.36 | | 3.49 (3.41) | | 10.05 | |
|  |  | 1.88 | 1.00 | 0 | 1.91 (0.29) | 0 | 0 | | 0.47 (0.40) | | 0.87 | |
|  |  | 1.83 | 0.79 | 0.07 | 1.63 (0) | 3.18 | 5.08 | | 0.08 (0) | | 0 | |
|  |  | 5.68 | 3.66 | 8.68 | 0 | 6.25 | 7.97 | | 0 | | 1.22 | |
| **Sat** | 1. R=cV 2. R=cV^α^ 3. R=cVe^αV^ | 0.42 | 1.91 (0.28) | 0 | 0 | 5.77 (5.33) | 11.81 | | 3.50 | | 2.78 (1.32) | |
|  |  | 0.35 | 1.63 (0) | 1.16 | 1.98 | 1.51 (1.06) | 0 | | 0.50 | | 1.69 (0.23) | |
|  |  | 0 | 2.34 (0.71) | 1.35 | 2.00 | 0.44 (0) | 1.77 | | 0 | | 1.46 (0) | |
|  | 1. R=c | 9.50 | 0 | 10.47 | 2.81 | 0 | 3.48 | | 4.47 | | 0 | |
| **Flood** | 1. R=cV 2. R=cV^α^ 3. R=cVe^αV^ | 0 | 5.27 | 0 | 4.39 | 0 | 0 | | 2.83 | | 8.62 | |
|  |  | 1.06 | 0 | 0.09 | 0.51 | 1.94 | 1.98 | | 0.33 | | 0 | |
|  |  | 0.50 | 0.93 | 0.52 | 0 | 1.86 | 2.00 | | 0 | | 1.72 | |
|  | 1. R=c | 1.99 | 0.54 | 8.85 | 11.44 | 6.06 | 1.50 | | 0.65 | | 15.24 | |
